# Differences in Sexually Transmitted Infections between the Precrisis Period (2000–2007) and the Crisis Period (2008–2014) in Granada, Spain

**DOI:** 10.3390/jcm8020277

**Published:** 2019-02-25

**Authors:** María Ángeles Pérez-Morente, María Teresa Sánchez-Ocón, Encarnación Martínez-García, Adelina Martín-Salvador, César Hueso-Montoro, Inmaculada García-García

**Affiliations:** 1 Facultad de Ciencias de la Salud, Universidad de Jaén, 23071 Jaén, Spain; mmorente@ujaen.es; 2 Complejo Hospitalario Virgen de las Nieves, 18014 Granada, Spain; maitesaoc@hotmail.com; 3 Facultad de Ciencias de la Salud, Universidad de Granada, 18016 Granada, Spain; emartinez@ugr.es (E.M.-G.); igarcia@ugr.es (I.G.-G.); 4 Facultad de Ciencias de la Salud, Universidad de Granada, 52005 Melilla, Spain; ademartin@ugr.es

**Keywords:** sexually transmitted diseases, public health, risk groups, communicable diseases, epidemiology

## Abstract

Objective: To analyze the difference in the prevalence of sexually transmitted infections (STIs) between two time periods (2000–2007 and 2008–2014, with the latter period characterized by the economic crisis), as well as determine differences in sociodemographic factors, clinical care, and risk indicators. Methods: This was a retrospective, observational, and analytical study, reviewing 1437 medical records of subjects attending a specialized center in the province of Granada (Spain) for consultation associated with the presence or suspicion of an STI between 2000–2014. Data were collected on variables relating to the research objective. A descriptive and bivariate statistical analysis was performed by multiple logistic regression. Results: In the analysis comparing the presence of STIs between the crisis and non-crisis periods, the percentage of positive diagnoses reached 56.6% compared to 43.4% negative diagnoses during the non-crisis period, while the percentages were 75.2% and 24.8%, respectively, during the crisis period. This difference was statistically significant (*p* < 0.001) with an odds ratio (OR) of 2.21 after adjusting for age, sex, days since last unprotected sexual intercourse, and partners in the last year. Conclusions: There are significant differences in the prevalence of STIs between the study periods, which is consistent with the reports of some authors regarding the effect of the financial crisis on these conditions; however, it is worth considering other aspects that might explain the differences.

## 1. Introduction

Sexually transmitted infections (STIs) pose a serious public health problem due to the number of people affected and the complications and associated consequences if they are not treated in a timely manner [1,2,3,4,5].

Since the mid-1990s in the European Union, there has been a steady increase in these diseases, especially acute bacterial infections, primarily affecting young people, ethnic minorities, and homosexuals [6]. The triggering factors include migratory movements, changes in risky sexual behaviors, the use of different drugs, and a decrease in safe sexual practices among homosexual men, among others [2].

In the context of globalization and economic crisis, some STIs that had been considered virtually eradicated in first-world countries, such as syphilis and gonorrhea, have reappeared [7]. Other diseases are also increasing, such as Acquired Immune Deficiency Syndrome (AIDS), hepatitis, chlamydia, genital herpes, human papillomavirus (HPV), and molluscum contagiosum.

According to the World Health Organization (WHO) [8], in addition to sexual behavior, the social or economic situation also increases the vulnerability of individuals to STIs. A systematic review of the impact of economic crises on the control and transmission of infectious diseases supports this conclusion [9], which is a hypothesis that has also been supported by other reports and research [10,11]. In Greece, for example, budget adjustments, along with the dismantling of one-third of all community prevention programs between 2009–2010, have been linked to an increase in Human Immunodeficiency Virus (HIV) and STIs, heroin use, and an increase in suicides, among other problems [11]. Additionally, in 2009, the WHO echoed similar effects associated with the financial crisis and global health [12].

Based on the above, the present study aims to analyze the difference in the prevalence of STIs between two time periods, 2000–2007 and 2008–2014, with the latter being characterized by the economic crisis. Additionally, differences were analyzed in relation to sociodemographic factors, clinical care, and risk indicators.

## 2. Materials and Methods 

A retrospective, observational, and analytical study was performed by reviewing medical records. The study was conducted at the Center for Sexually Transmitted Diseases and Sexual Orientation of San Juan de Dios Hospital in Granada. The data were retrieved from medical records of subjects attending the center for consultation associated with the presence or suspicion of transmission of an STI. The medical record included four options in this regard (symptomatology, control, follow-up of contacts, and HIV), and medical records in which one of these options was checked were selected. The other two options included in the medical record were voluntary interruption of pregnancy (VIP) and family planning, which were discarded. Medical records corresponding to adults with no cognitive impairment at the time of contacting the center (verified when accessing the medical record) were reviewed. The study period was from 2000 to 2014, and the periods of 2000–2007 and 2008–2014 were compared. These periods are similar in terms of the number of years analyzed, but differ based on the impact of the crisis during the second period [13].

The sample size was determined to allow for detecting differences in the basic variables of STI presentation in subjects, for whom a new medical record was opened. This calculation was carried out to detect differences in a binary variable with the aim of detecting a 20% difference in two years, with 80% power, provided that the test error was α = 5%; therefore, a large sample size was needed. The sample size that was needed to detect this difference was 97 medical records per year, resulting in an overall sample size of 97 × 15 = 1455 medical records for the 15 years under consideration. Initially, 1510 medical records were reviewed, and after the data were cleaned for the final statistical analysis, the sample was reduced to 1437, which implies a high inclusion rate (98.8%) with respect to the sample size calculation.

The sample was extracted from the new medical records archived for each year using the first and last medical record number of that year and selecting a sample of each year by systematic random sampling. When the selected medical record did not meet the inclusion criteria described above, the immediately prior medical record was selected; when the criteria were not met, the medical record immediately after the initial record was selected. If the inclusion criteria were not met in both cases, the selection continued backward and forward until a medical record meeting the inclusion criteria was obtained. The interval was set to 15 as the most repeated value in the series.

An ad hoc data collection sheet was prepared based on the study variables. Then, a computerized database was designed to contain the information compatible with the statistical analysis computer program that was used. The data collection was carried out in person at the center using paper medical records by three people who were previously trained to ensure a homogeneous and consensual process. An initial pilot study was conducted on 110 medical records to refine the data collection sheet and clarify doubts regarding some variables. The three people who took part in the data collection had a college education in the health sciences. The variables are described in Table S1 (Supplementary Materials).

The variables studied were grouped into different categories: sociodemographic characteristics, characteristics of the clinical care received, and risk indicators for STIs; the variables of STI diagnosis and crisis were considered separately from the other categories.

For the statistical analysis, the mean, standard deviation, median, and interquartile range for continuous variables were calculated, while the absolute frequency and percentage were used for categorical variables. To compare the variables of the two study periods, nonparametric hypothesis contrast tests were used due to the non-normality of the continuous variables. Thus, the Mann–Whitney U test, Kruskal–Wallis test, and Spearman correlation were employed. To compare categorical variables, contingency tables were created and the chi-squared test (χ²) was carried out; when this could not be applied, the generalization of Fisher’s exact test was used. These calculations were performed with the statistical software IBM SPSS version 22.

To complete the analysis, a multiple logistic regression analysis was performed, taking STI diagnosis as an outcome variable and crisis as the main independent variable. The association was adjusted with confounding variables. To select the confounding variables, the number of included variables needed to be adapted to the recommended requirement in multivariate analysis in order to have at least 15 cases for each variable included in the model. Here, the smallest number of the variables studied was taken as a reference, considering that, for some variables, there was a high percentage of missing data. For each variable included in the model, the odds ratio (OR) with a 95% CI was calculated. Once the model was generated, the fit conditions were tested. Collinearity between variables was investigated by calculating the variance inflation factor (VIF); considering the absence of collinearity with VIF < 2.5, the linearity of the dependent variable was tested with the continuous variables included in the model, and the calibration was determined by the Hosmer–Lemeshow goodness-of-fit test, which is reflected by the absence of significant differences (*p* > 0.05) between the observed and expected values according to the model. Finally, discrimination was determined from the value of the area under the receiver operating characteristic (ROC) curve, which was considered to be adequate at >0.70. Several models with different variables were tested, and the model shown in the results was validated and fulfilled the abovementioned fit criteria. The calculations were performed with the software R commander, R version 3.2.2 (https://www.r-project.org/, Spanish R-UCA Project, http://knuth.uca.es/R).

In all of the analyses, *p* < 0.05 was considered to be statistically significant.

This study was approved by both the Biomedical Research Ethics Committee of the Granada province and the Management Directorate of the Granada Metropolitan Health District, which oversaw the center conducting the research. The data were treated with the utmost confidentiality according to the 13 December Organic Law 15/1999 on the Protection of Personal Data.

## 3. Results

The results show that in both periods, the populations were homogenous in terms of age, employment, and marital status, finding statistically significant differences by sex, citizenship, occupation, and educational level (Table 1).

Regarding the clinical care received, significant differences were found in the number of subsequent visits, reason for consultation, and prior consultation. There were no differences in the number of new subsequent episodes (Table 2).

Regarding risk indicators, there were differences between the populations in the time since last unprotected sexual intercourse, age of the first sexual intercourse, sexual behavior, and sexual contact with sex workers. The population was homogeneous in the other variables (Table 3).

When analyzing the presence of STIs between the crisis and non-crisis periods, it was found that during the non-crisis period, the percentage of positive diagnoses was 56.6% (*n* = 201) compared to 43.4% (*n* = 154) for negative diagnoses; during the crisis, the percentages were 75.2% (*n* = 243) and 24.8% (*n* = 80), respectively. This difference was statistically significant (χ² = 25.922, df = 1, *p* < 0.001), with an OR (crisis/non-crisis) of 2.33. In the regression model generated, this association was adjusted for the following variables: age, sex, days since last unprotected sexual intercourse, and partners in the last year (Table 4).

**Figure 1:**
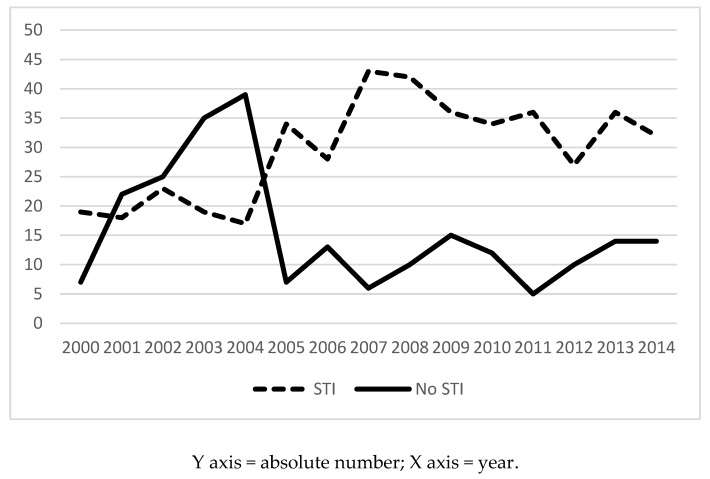
Prevalence of STI diagnosis during the study period.

Figure 1 shows the progress of positive and negative diagnoses during the study period. Table 5 shows the comparison of STI diagnoses between the two analyzed periods.

## 4. Discussion

Regarding the sociodemographic profile, more men than women attended the center during the crisis. In both periods, more Spanish citizens than immigrants attended the center; however, during the crisis, the percentage of immigrants was lower, which may be because after the economic crisis, many immigrants living in Spain returned to their countries of origin in the face of worsening employment and economic conditions. These findings are consistent with the study of the EPI–VIH group [14]. Notably, during the crisis, there was an increase in the percentage of subjects with higher academic levels attending the center.

During the crisis, the percentage of sex workers attending the center decreased, which is consistent with the study of the EPI–VIH group [14]. In our study, most of these sex workers were women who were immigrants, and were probably in an irregular administrative situation. This could lead—because of the restrictive measures in access to public health promulgated by the Royal Decree-Law 16/2012—to the worsening of an already complicated situation for access to health and/or social services [15,16]. Regarding differences in the clinical care received, the study found that there was a greater number of subsequent visits during the crisis. This is consistent with the finding that the percentage of positive STI diagnoses was almost 20 points higher during this period, justifying a more intense follow-up of patients, which is a finding that was also emphasized in the study of the EPI–VIH group [14]. In contrast, the percentage of prior visits was lower during the crisis, which could be explained by difficulties of access to the health system during the crisis, which excessively affected disadvantaged social classes and ethnic minorities [17], which were groups that were represented in our study.

There was an increase in the percentage of consultations related to HIV during the crisis. Although HIV was not one of the infections that increased the most during the crisis, this increase may indicate more risky behaviors in the crisis period versus the non-crisis period.

In the analysis of risk indicators, a decrease was found in the time since last unprotected sexual intercourse during the crisis. Some authors suggested that since 2007, there has been a decrease in the use of contraceptives and an absence of protective measures against STIs in a fifth of occasional or sporadic sexual encounters. They suggest that difficulties of accessing contraception would be related to economic problems by hindering an individual’s ability to pay for it [18]. Hence, during the crisis, the economic difficulties generated by high unemployment and precarious work could explain lower condom use [19], and therefore a decreased time since the last unprotected sexual intercourse, consequently increasing the risk of contagion of venereal diseases.

Regarding sexual behavior, more homosexual and fewer heterosexual individuals went to the center during the crisis, which is a finding consistent with the results of the EPI–VIH group [14]. Finally, during the crisis, there was a decrease in the percentage of subjects having sexual contact with sex workers, perhaps because the worsening economic conditions could have led to a lower demand for these sexual services.

By examining the differences in STI diagnoses between the two analyzed periods, a nearly 20-point increase in positive diagnoses was found during the crisis period compared to the non-crisis period. From the ORs in the regression model, it can be deduced that under equal conditions for age, sex, days since last unprotected sexual intercourse, and number of partners in the last year, the crisis period had a higher probability for the appearance of STIs than the non-crisis period, and was 2.21-fold higher during the crisis.

Specifically, the most prevalent STI in our study was HPV, whose number of diagnosed cases increased during the crisis, followed by gardnerella, molluscum contagiosum, syphilis, gonorrhea and HIV. The fact that HPV was the most diagnosed STI in our study coincides with the study on STIs in Andalusia [20], in which this infection was the most common in 2009, followed by Chlamydia trachomatis, gonorrhea, syphilis, genital herpes, and HIV. If we consider the recommendation to introduce routine vaccination against HPV in girls between 11–14 years of age (approved by the Interterritorial Council in 2007, establishing vaccination at 14 years [21]), then the observed increase must be interpreted in a context in which preventive measures are being intensified, which may indicate underdiagnosis in the non-crisis period.

Regarding the other diagnosed STIs, although the most reported STI in Europe is caused by Chlamydia trachomatis [22,23], it was practically non-existent in our study. The syphilis and gonorrhea data in the period from 1995–2013 [24] show a continuous increase in the incidence of these conditions, and since 2004, the rate of syphilis has surpassed the rate of gonorrhea. According to data from the Microbiological Information System [25], since 2012, there has been an increase in the number of reported infections. For HIV, a slight increase was observed during the crisis, and in the period of 2000–2013 [14], there was an increase in the prevalence of HIV in Spain since 2006.

In conclusion, apart from the disaggregated STI analyses, there were significant differences in the prevalence of STIs between the study periods, which is consistent with the reports of other authors regarding the effect of the financial crisis on these diseases [26,27].

Obviously, we should be cautious with this result due to some conditions that have already been discussed and due to the limitations of the study. For example, the results cannot be generalized, since the study includes subjects who, by the mere fact of going to this center, have engaged in risk behaviors. Another limitation is related to the study design that was used. Despite having analyzed a broad time series and having a large number of cases, due to the cross-sectional design, the associations found should be considered causal hypotheses, which should be verified with more complex study designs.

The findings of this research suggest some actions to be taken. For example, public policies should focus on three areas. First, policies should promote and develop an active search of cases among the contacts of an individual with an STI as a fundamental measure to interrupt the chain of transmission and prevent further infection. Second, they should prioritize the fight against a late diagnosis of these infections, since early detection and treatment is one of the most cost-effective interventions. Third, they should promote the development of more studies such as this, which would contribute to normalize STIs and make them visible, thereby reducing the discrimination and stigmatization associated with them, as this is one of the main health care access barriers. In this sense, not only does stigmatization and discrimination create a barrier to approaching these diseases, but the lack of knowledge of the different STIs among the population, especially among young people, also contributes to the increase in and spread of STIs, as affirmed by several recent European studies [28,29].

## 5. Conclusions

There are significant differences in the prevalence of STIs between the study periods, which is consistent with the reports of some authors regarding the effect of the financial crisis on these conditions; however, it is worth considering other aspects that might explain the differences.

## Figures and Tables

**Table 1:** Sociodemographic Characteristics during the Crisis vs. Non-crisis Periods.

Variable	Crisis			Non-Crisis			Test	
	*n*	Mean	Sd	*n*	Mean	Sd	U ^a^	*p*
Age (*n* = 1437)	690	28.99	9.040	747	28.92	8.780	257,509	0.979
**Variables**	***n***		**%**	***n***		**%**	**χ²(df) ^b^**	***p***
Sex (*n* = 1437)							6.941 (1)	0.008
Male	397		51.2%	378		48.8%		
Female	293		44.3%	369		55.7%		
Citizenship (*n* = 1421)							7.739 (1)	0.005
Spanish	530		50.6%	517		49.4%		
Immigrant	158		42.2%	216		57.8%		
Occupation (*n* = 1343)							8.147 (2)	0.017
Sex worker	61		40.9%	88		59.1%		
Other occupation	353		53.4%	308		46.6%		
Student	261		49%	272		51%		
Employment (*n* = 1298)							3.900 (3)	0.272
Active	265		47.3%	295		52.7%		
Unemployed	107		54.9%	88		45.1%		
Retired	5		38.5%	8		61.5%		
Student	261		49.2%	269		50.8%		
Educational level (*n* = 1350)							25.645 (4)	<0.001
Without education	7		58.3%	5		41.7%		
Primary	88		40%	132		60%		
Secondary	122		41.1%	175		58.9%		
Vocational training	87		59.6%	59		40.4%		
Higher	357		52.9%	318		47.1%		
Marital status (*n* = 1426)							7.150 (3)	0.067
Single	579		49.3%	596		50.7%		
Married/Domestic partners	65		38.7%	103		61.3%		
Separated/Divorced	39		51.3%	37		48.7%		
Widower	4		57.1%	3		42.9%		

^a^ Mann–Whitney U-test (Z = −0.26). Me (Interquartile Range (IQR)): Yes = 27 (23–33); No = 26 (23–32). ^b^ Pearson’s chi-square.

**Table 2:** Clinical Care during the Crisis vs. Non-Crisis Periods.

Variable	Crisis			Non-Crisis			Test	
	*n*	Mean	Sd	*n*	Mean	Sd	U ^a^	*p*
Number of subsequent visits (*n* = 1432)	687	1.22	1.048	745	1	1.064	221,185.5	<0.001
Number of subsequent new episodes (*n* = 1432)	685	0.42	0.954	747	0.41	0.929	252,943	0.621
**Variable**	***n***		**%**	***n***		**%**	**χ²(df) ^b^**	***p***
Reason for consultation (*n* = 1437)							42.884 (3)	<0.001
Symptoms	212		45.8%	251		54.2%		
Control	10		21.3%	37		78.7%		
Follow-up of contacts	0		0%	25		100%		
HIV	468		51.9%	434		48.1%		
Prior consultation (*n* = 1059)							7.155 (1)	0.007
Yes	202		53.6%	175		46.4%		
No	423		62%	259		38%		

^a^ Mann–Whitney U-test (Z subsequent visits = −4.856; Z new episodes = −0.465). Subsequent visits, Me (IQR): Yes = 1 (1–1); No = 1 (0–1)/New episodes, Me (IQR): Yes = 0 (0–0); No = 0 (0–1). ^b^ Pearson’s chi-square.

**Table 3:** Risk Indicators during the Crisis vs. Non-cCisis Periods.

Variable	Crisis			Non-Crisis			Test	
	*n*	Mean	Sd	*n*	Mean	Sd	U ^a^	*p*
Days since last unprotected sexual intercourse (*n* = 933)	504	2.52	0.873	429	2.79	0.943	93,602.5	<0.001
Partners in the last month (*n* = 1355)	676	1.58	1.223	679	1.67	1.328	223,624.5	0.279
Partners in the last year (*n* = 1339)	668	2.72	1.759	671	2.79	1.949	222,176	0.778
Sex life (*n* = 379)	212	1.74	0.874	167	1.91	0.924	16,062	0.092
Age at first sexual intercourse (*n* = 840)	498	17.79	2.897	342	18.02	3.240	75,834	0.006
**Variable**	***n***		**%**	***n***		**%**	**χ²(df) ^b^**	***p***
Sexual behavior (*n* = 1408)							41.179 (2)	<0.001
Heterosexual	515		44.1%	652		55.9%		
Bisexual	36		67.9%	17		32.1%		
Homosexual	125		66.5%	63		33.5%		
Regular partner (*n* = 1336)							1.907 (1)	0.167
Yes	414		48.6%	437		51.4%		
No	255		52.6%	230		47.4%		
Sexual intercourse with sex workers (*n* = 564)							6.905 (1)	0.009
Yes	53		41.1%	76		58.9%		
No	236		54.3%	199		45.7%		
Regular partner with symptoms (*n* = 461)							0.512 (1)	0.474
Yes	111		62.4%	67		37.6%		
No	167		59%	116		41%		
Drug use (*n* = 750)							0.000 (1)	0.988
Yes	148		55.4%	119		44.6%		
No	268		55.5%	215		44.5%		
Prior STIs (*n* = 1164)							0.355 (1)	0.551
Yes	409		44.7%	507		55.3%		
No	116		46.8%	132		53.2%		

^a^ Mann–Whitney U-test (Z condom days = −3.811; Z partners month = −1.083; Z partners year = −0.282; Z sexual life = −1.687; Z age at intercourse = −2.728). Condom days, Me (IQR): Yes = 2 (2–3); No = 3 (2–3)/Partners month, Me (IQR): Yes = 1 (1–1); No = 1 (1–2)/Partners year, Me (IQR): Yes = 2 (1–4); No = 2 (1–3)/Sex life, Me (IQR): Yes = 1 (1–2.75); No = 2 (1–3)/Age at first intercourse, Me-IQR: Yes = 17 (16–18); No = 18 (16–19). ^b^ Pearson’s chi-square.

**Table 4:** Logistic Regression Model for Sexually Transmitted Infection (STI) Diagnosis.

Variable	OR (95% CI)	*p*	VIF ^a^
Age	0.98 (0.95–1.004)	0.112	1.05
Sex		0.003	1.10
Female	2.21 (1.31–3.80)		
Male	Ref.		
Days since last unprotected sexual intercourse	0.80 (0.61–1.05)	0.123	1.05
Partners in the last year	1.27 (1.10–1.48)	0.001	1.00
Crisis		0.001	1.06
Yes	2.21 (1.37–3.59)		
No	Ref.		

^a^ Variance Inflation Factor (VIF). Calibration through the Hosmer–Lemeshow goodness-of-fit test: X-squared = 6.976, df = 8, *p*-value = 0.539. Discrimination according to the receiver operating characteristic (ROC) curve: area under the ROC curve with a value of 0.721 (95% CI = 0.668–0.771).

**Table 5:** STI Comparison between the Crisis Period vs. Non-Crisis Periods.

Diagnosis	Crisis		Non-Crisis	
	*n*	%	*n*	%
Human Papilloma Virus (HPV)	103	59.9%	69	40.1%
Gonorrhea	12	54.5%	10	45.5%
Gardnerella	13	61.9%	8	38.1%
Syphilis	13	56.5%	10	43.5%
Candida	22	37.3%	37	62.7%
Molluscum contagiosum	18	56.3%	14	43.8%
Herpes Simplex Virus (HSV)	10	47.6%	11	52.4%
Human Immunodeficiency Virus (HIV)	6	54.5%	5	45.5%
Trichomoniasis	1	25%	3	75%
Chlamydia	0	0%	1	100%
Hepatitis C Virus (HCV)	0	0%	1	100%

## Supplementary Materials

The following are available online at https://www.mdpi.com/2077-0383/8/2/277/s1, Table S1: Detailed description of the study variables.
