# Supplementary material for: Differences in Sexually Transmitted Infections between the Precrisis Period (2000–2007) and the Crisis Period (2008–2014) in Granada, Spain
Source: J Clin Med. 2019 Feb 25;8(2):277. doi: 10.3390/jcm8020277 (PMC6406728; doi:10.3390/jcm8020277)
Supplement: Supplementary file 1 [file jcm-08-00277-s001.pdf]

Table S1. Detailed description of the study variables.

Variable	Type	Medical Record	Categories / Transformations
Year of consultation	Categorical polychotomous	Yes	-
Crisis		No	This variable was obtained from the year of consultation, establishing two categories: Non-Crisis (including medical records whose year of consultation corresponded to the period 2000–2007) and Crisis (medical records corresponding to the period 2008–2014). These two periods were established because 2008 is the year in which the financial crisis began in Spain.
Sex	Categorical dichotomous	Yes	Male, Female.
Age	Continuous	Yes	-
Citizenship	Categorical polychotomous / dichotomous	Yes	For statistical analysis, citizenship was transformed into a dichotomous variable to differentiate Spanish citizenship from the other identified nationalities, which were grouped in a category called "immigrant".
Occupation	Categorical polychotomous	Yes	For statistical analysis, occupation was transformed into a variable with three categories: Sex worker/former sex worker, Student, and Other occupation/unpaid occupation.
Employment	Categorical polychotomous	Yes	Active, Unemployed, Retired, Student.
Educational level	Categorical polychotomous	Yes	No studies, Primary/Elementary/General Basic Education (EGB, Spanish acronym), Secondary/Baccalaureate, Vocational Training, College.
Civil status	Categorical polychotomous	Yes	Single, Married/Domestic partner, Separated/Divorced/Widowed.
Reason for consultation	Categorical polychotomous	Yes	Symptoms, Control, Follow-up of contacts, HIV.
Prior consultation	Categorical dichotomous	Yes	Yes, No.
Who provided prior consultation	Categorical polychotomous	Yes	When the answer to the previous variable was affirmative, the type of department where the consultation took place was recorded, showing great heterogeneity. For statistical analysis, this variable was transformed into the following categories: Specialist consultation, Primary care, Emergencies, Private clinic, Specific STI center, Social services, Other.
Number of subsequent visits	Continuous	No	The data corresponded to the number of visits resulting from the first reason for consultation, which were obtained by reviewing the follow-up history appearing in the medical record, organized by date. Each date was preceded by a number so that zero corresponded to the follow-up record related to the first reason for consultation.
Number of subsequent new episodes	Continuous	No	The procedure to determine the data of this variable was similar to that described above. Here, when the episode differed from the initial one, the professionals coded the follow-up date with a number different from zero, so that one corresponded with a new episode, and so on.
STI diagnosis	Categorical dichotomous	No	Yes, No. This variable was created from the clinical findings recorded in the history of each subject.
Sexual behavior	Categorical polychotomous	Yes	Heterosexual, Bisexual, Homosexual, Transsexual.
Regular partner	Categorical dichotomous	Yes	Yes, No.
Days since last unprotected sexual intercourse	Categorical polychotomous / Continuous	Yes	Never, Less than one month, one to six months, six to 12 months, and over 12 months. For statistical analysis, sexual intercourse was transformed into a continuous variable, with the scores ranging from one to five, corresponding to the categories mentioned above. This scoring system measured the intensity of condom use; low scores reported inconsistent and infrequent condom use, and high scores reported consistent use.
Sexual partners in the last month	Categorical polychotomous / Continuous	Yes	0–1, 2, 3–5, over 5, Prostitution. As in the previous case, the variable was transformed into a continuous variable, also with scores ranging from one to five.
Sexual partners in the last year	Categorical polychotomous / Continuous	Yes	0–1, 2, 3–5, 6–10, 11–20, over 20, Prostitution. This variable also became a continuous scale variable, in this case with scores ranging from one to seven.

Sex life	Categorical polychotomous / Continuous	Yes	0–10, 10–20, over 20. Following the trend of the previous variables, this variable also became a continuous scale variable for the analysis, with scores ranging from one to three.
Contact with a sex worker	Categorical dichotomous	Yes	Yes, No.
Frequency of contact with a sex worker	Categorical polychotomous	Yes	Usually, Occasionally, Once.
Suspicious contact	Categorical dichotomous	Yes	Yes, No.
Usual partner with symptoms	Categorical dichotomous	Yes	Yes, No.
Drug use	Categorical dichotomous	Yes	Yes, No.
Frequency of drug use	Categorical polychotomous	Yes	Usually, Occasionally, Not currently.
Parenteral Drug Administration (PDA)	Categorical dichotomous	Yes	Yes, No.
Age at first sexual intercourse	Continuous	Yes	-
